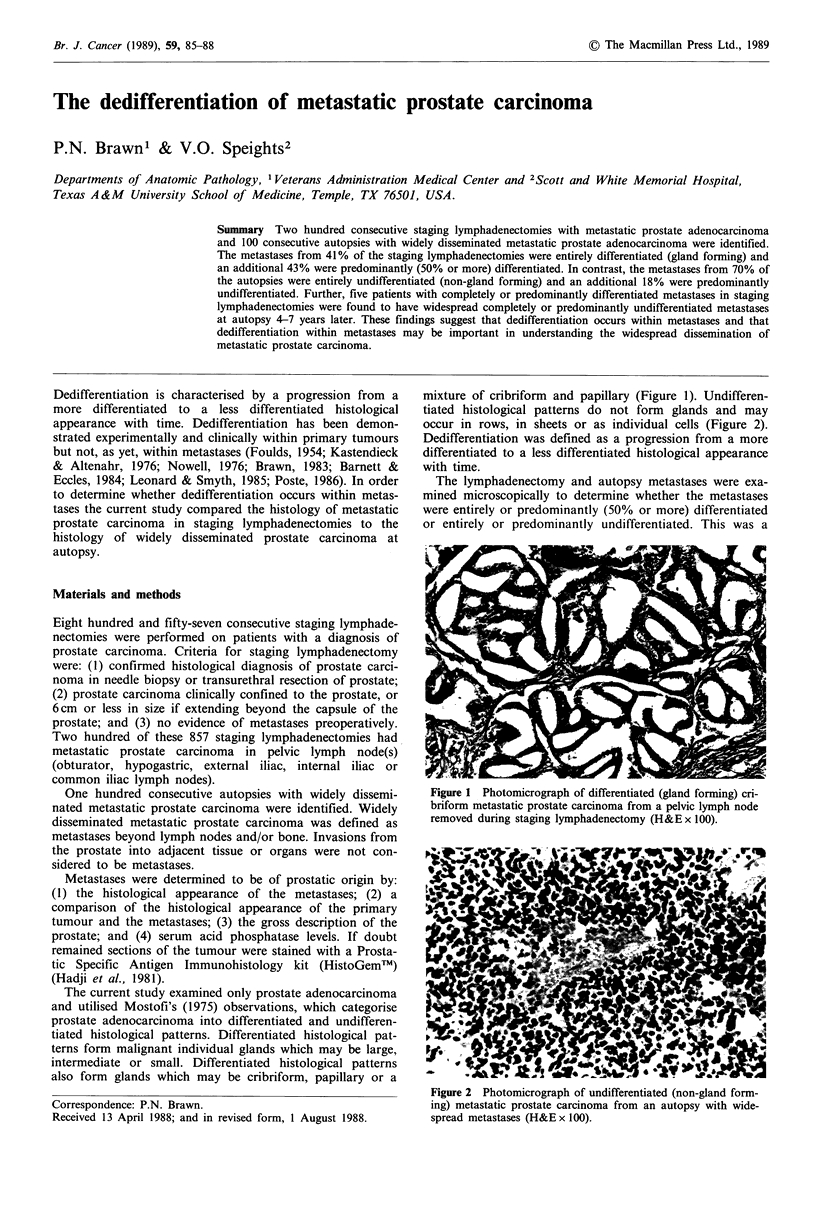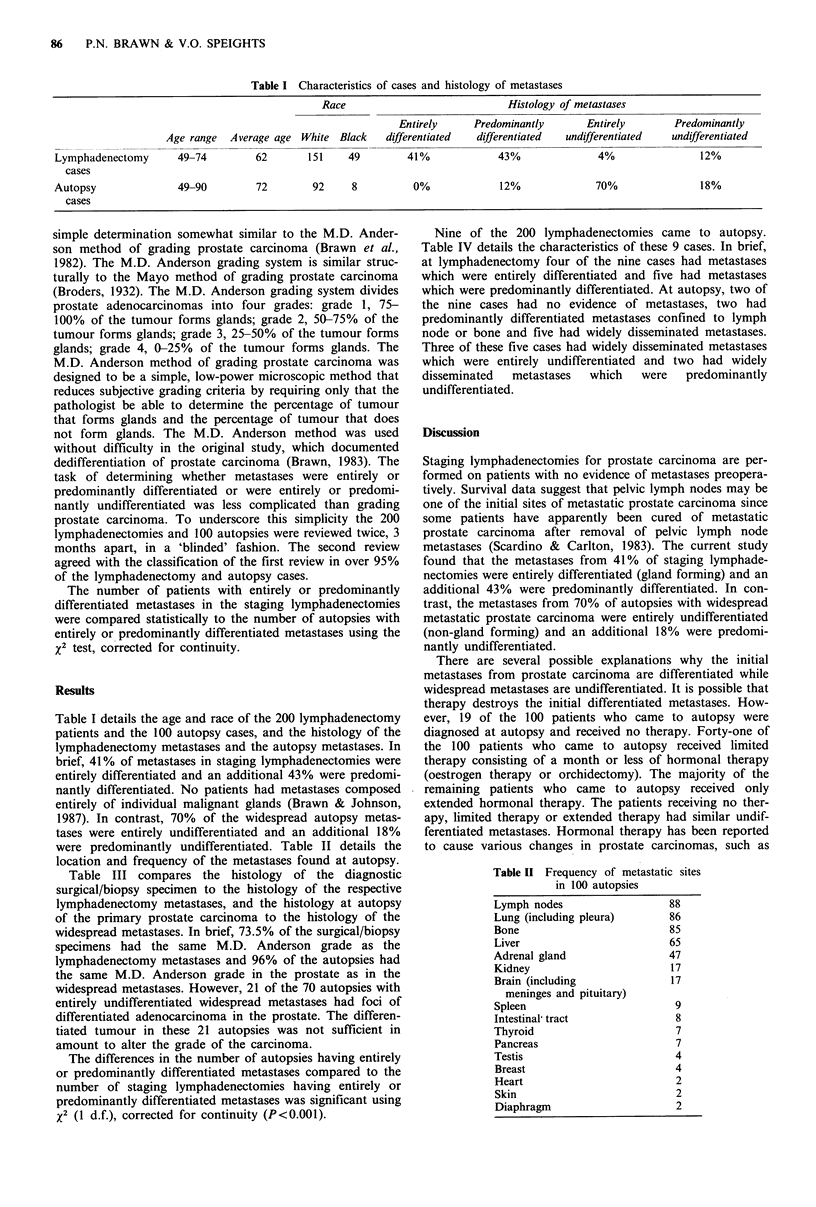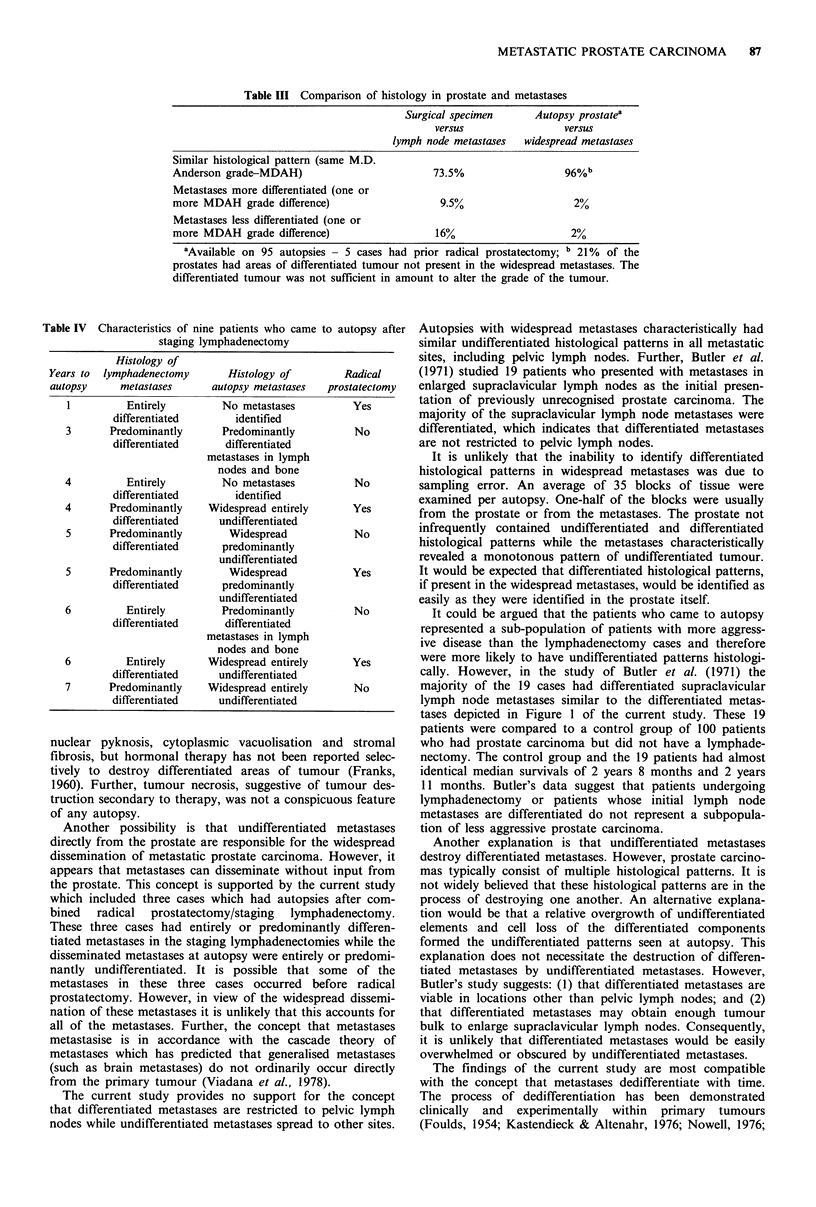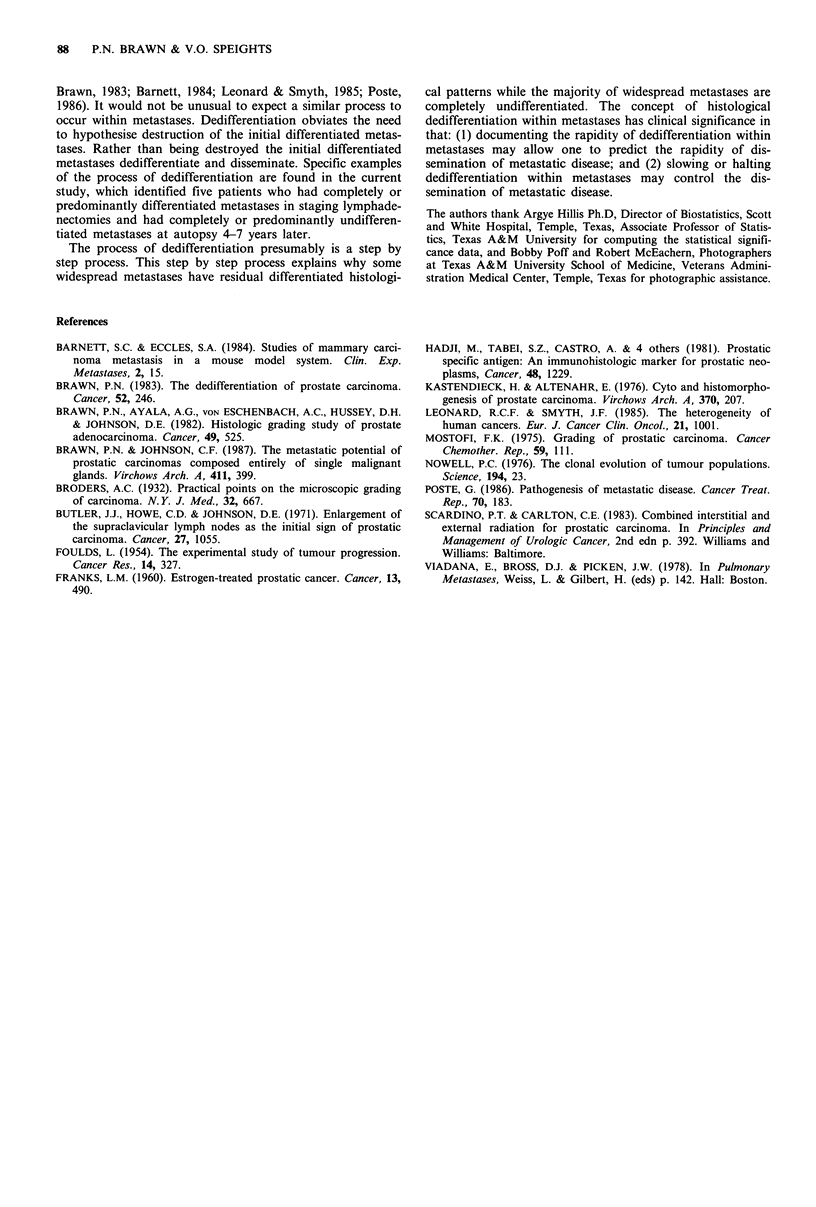# The dedifferentiation of metastatic prostate carcinoma.

**DOI:** 10.1038/bjc.1989.16

**Published:** 1989-01

**Authors:** P. N. Brawn, V. O. Speights

**Affiliations:** Department of Anatomic Pathology, Veterans Administration Medical Center, Temple, TX 76501.

## Abstract

**Images:**


*Br. J. Cancer* (1989), **59**, 85–88 © The Macmillan Press Ltd., 1989

### The dedifferentiation of metastatic prostate carcinoma

P.N. Brawn[1] & V.O. Speights[2]

*Departments of Anatomic Pathology, [1]Veterans Administration Medical Center and [2]Scott and White Memorial Hospital, Texas A&M University School of Medicine, Temple, TX 76501, USA.*

**Summary** Two hundred consecutive staging lymphadenectomies with metastatic prostate adenocarcinoma and 100 consecutive autopsies with widely disseminated metastatic prostate adenocarcinoma were identified. The metastases from 41% of the staging lymphadenectomies were entirely differentiated (gland forming) and an additional 43% were predominantly (50% or more) differentiated. In contrast, the metastases from 70% of the autopsies were entirely undifferentiated (non-gland forming) and an additional 18% were predominantly undifferentiated. Further, five patients with completely or predominantly differentiated metastases in staging lymphadenectomies were found to have widespread completely or predominantly undifferentiated metastases at autopsy 4–7 years later. These findings suggest that dedifferentiation occurs within metastases and that dedifferentiation within metastases may be important in understanding the widespread dissemination of metastatic prostate carcinoma.

Dedifferentiation is characterised by a progression from a more differentiated to a less differentiated histological appearance with time. Dedifferentiation has been demonstrated experimentally and clinically within primary tumours but not, as yet, within metastases (Foulds, 1954; Kastendieck & Altenahr, 1976; Nowell, 1976; Brawn, 1983; Barnett & Eccles, 1984; Leonard & Smyth, 1985; Poste, 1986). In order to determine whether dedifferentiation occurs within metastases the current study compared the histology of metastatic prostate carcinoma in staging lymphadenectomies to the histology of widely disseminated prostate carcinoma at autopsy.

### Materials and methods

Eight hundred and fifty-seven consecutive staging lymphadenectomies were performed on patients with a diagnosis of prostate carcinoma. Criteria for staging lymphadenectomy were: (1) confirmed histological diagnosis of prostate carcinoma in needle biopsy or transurethral resection of prostate; (2) prostate carcinoma clinically confined to the prostate, or 6 cm or less in size if extending beyond the capsule of the prostate; and (3) no evidence of metastases preoperatively. Two hundred of these 857 staging lymphadenectomies had metastatic prostate carcinoma in pelvic lymph node(s) (obturator, hypogastric, external iliac, internal iliac or common iliac lymph nodes).

One hundred consecutive autopsies with widely disseminated metastatic prostate carcinoma were identified. Widely disseminated metastatic prostate carcinoma was defined as metastases beyond lymph nodes and/or bone. Invasions from the prostate into adjacent tissue or organs were not considered to be metastases.

Metastases were determined to be of prostatic origin by: (1) the histological appearance of the metastases; (2) a comparison of the histological appearance of the primary tumour and the metastases; (3) the gross description of the prostate; and (4) serum acid phosphatase levels. If doubt remained sections of the tumour were stained with a Prostatic Specific Antigen Immunohistology kit (HistoGem™) (Hadji *et al.*, 1981).

The current study examined only prostate adenocarcinoma and utilised Mostofi's (1975) observations, which categorise prostate adenocarcinoma into differentiated and undifferentiated histological patterns. Differentiated histological patterns form malignant individual glands which may be large, intermediate or small. Differentiated histological patterns also form glands which may be cribriform, papillary or a mixture of cribriform and papillary (Figure 1). Undifferentiated histological patterns do not form glands and may occur in rows, in sheets or as individual cells (Figure 2). Dedifferentiation was defined as a progression from a more differentiated to a less differentiated histological appearance with time.

The lymphadenectomy and autopsy metastases were examined microscopically to determine whether the metastases were entirely or predominantly (50% or more) differentiated or entirely or predominantly undifferentiated. This was a

**Figure 1** Photomicrograph of differentiated (gland forming) cribriform metastatic prostate carcinoma from a pelvic lymph node removed during staging lymphadenectomy (H&E × 100).

**Figure 2** Photomicrograph of undifferentiated (non-gland forming) metastatic prostate carcinoma from an autopsy with widespread metastases (H&E × 100).

Correspondence: P.N. Brawn.
Received 13 April 1988; and in revised form, 1 August 1988.

**Table I** Characteristics of cases and histology of metastases

| | | | Race | | Histology of metastases | | | |
|---|---|---|---|---|---|---|---|---|
| | *Age range* | *Average age* | *White* | *Black* | *Entirely differentiated* | *Predominantly differentiated* | *Entirely undifferentiated* | *Predominantly undifferentiated* |
| Lymphadenectomy cases | 49–74 | 62 | 151 | 49 | 41% | 43% | 4% | 12% |
| Autopsy cases | 49–90 | 72 | 92 | 8 | 0% | 12% | 70% | 18% |

simple determination somewhat similar to the M.D. Anderson method of grading prostate carcinoma (Brawn *et al.*, 1982). The M.D. Anderson grading system is similar structurally to the Mayo method of grading prostate carcinoma (Broders, 1932). The M.D. Anderson grading system divides prostate adenocarcinomas into four grades: grade 1, 75–100% of the tumour forms glands; grade 2, 50–75% of the tumour forms glands; grade 3, 25–50% of the tumour forms glands; grade 4, 0–25% of the tumour forms glands. The M.D. Anderson method of grading prostate carcinoma was designed to be a simple, low-power microscopic method that reduces subjective grading criteria by requiring only that the pathologist be able to determine the percentage of tumour that forms glands and the percentage of tumour that does not form glands. The M.D. Anderson method was used without difficulty in the original study, which documented dedifferentiation of prostate carcinoma (Brawn, 1983). The task of determining whether metastases were entirely or predominantly differentiated or were entirely or predominantly undifferentiated was less complicated than grading prostate carcinoma. To underscore this simplicity the 200 lymphadenectomies and 100 autopsies were reviewed twice, 3 months apart, in a 'blinded' fashion. The second review agreed with the classification of the first review in over 95% of the lymphadenectomy and autopsy cases.

The number of patients with entirely or predominantly differentiated metastases in the staging lymphadenectomies were compared statistically to the number of autopsies with entirely or predominantly differentiated metastases using the $\chi^2$ test, corrected for continuity.

### Results

Table I details the age and race of the 200 lymphadenectomy patients and the 100 autopsy cases, and the histology of the lymphadenectomy metastases and the autopsy metastases. In brief, 41% of metastases in staging lymphadenectomies were entirely differentiated and an additional 43% were predominantly differentiated. No patients had metastases composed entirely of individual malignant glands (Brawn & Johnson, 1987). In contrast, 70% of the widespread autopsy metastases were entirely undifferentiated and an additional 18% were predominantly undifferentiated. Table II details the location and frequency of the metastases found at autopsy.

Table III compares the histology of the diagnostic surgical/biopsy specimen to the histology of the respective lymphadenectomy metastases, and the histology at autopsy of the primary prostate carcinoma to the histology of the widespread metastases. In brief, 73.5% of the surgical/biopsy specimens had the same M.D. Anderson grade as the lymphadenectomy metastases and 96% of the autopsies had the same M.D. Anderson grade in the prostate as in the widespread metastases. However, 21 of the 70 autopsies with entirely undifferentiated widespread metastases had foci of differentiated adenocarcinoma in the prostate. The differentiated tumour in these 21 autopsies was not sufficient in amount to alter the grade of the carcinoma.

The differences in the number of autopsies having entirely or predominantly differentiated metastases compared to the number of staging lymphadenectomies having entirely or predominantly differentiated metastases was significant using $\chi^2$ (1 d.f.), corrected for continuity ($P<0.001$).

Nine of the 200 lymphadenectomies came to autopsy. Table IV details the characteristics of these 9 cases. In brief, at lymphadenectomy four of the nine cases had metastases which were entirely differentiated and five had metastases which were predominantly differentiated. At autopsy, two of the nine cases had no evidence of metastases, two had predominantly differentiated metastases confined to lymph node or bone and five had widely disseminated metastases. Three of these five cases had widely disseminated metastases which were entirely undifferentiated and two had widely disseminated metastases which were predominantly undifferentiated.

### Discussion

Staging lymphadenectomies for prostate carcinoma are performed on patients with no evidence of metastases preoperatively. Survival data suggest that pelvic lymph nodes may be one of the initial sites of metastatic prostate carcinoma since some patients have apparently been cured of metastatic prostate carcinoma after removal of pelvic lymph node metastases (Scardino & Carlton, 1983). The current study found that the metastases from 41% of staging lymphadenectomies were entirely differentiated (gland forming) and an additional 43% were predominantly differentiated. In contrast, the metastases from 70% of autopsies with widespread metastatic prostate carcinoma were entirely undifferentiated (non-gland forming) and an additional 18% were predominantly undifferentiated.

There are several possible explanations why the initial metastases from prostate carcinoma are differentiated while widespread metastases are undifferentiated. It is possible that therapy destroys the initial differentiated metastases. However, 19 of the 100 patients who came to autopsy were diagnosed at autopsy and received no therapy. Forty-one of the 100 patients who came to autopsy received limited therapy consisting of a month or less of hormonal therapy (oestrogen therapy or orchidectomy). The majority of the remaining patients who came to autopsy received only extended hormonal therapy. The patients receiving no therapy, limited therapy or extended therapy had similar undifferentiated metastases. Hormonal therapy has been reported to cause various changes in prostate carcinomas, such as

**Table II** Frequency of metastatic sites in 100 autopsies

| | |
|---|---|
| Lymph nodes | 88 |
| Lung (including pleura) | 86 |
| Bone | 85 |
| Liver | 65 |
| Adrenal gland | 47 |
| Kidney | 17 |
| Brain (including meninges and pituitary) | 17 |
| Spleen | 9 |
| Intestinal tract | 8 |
| Thyroid | 7 |
| Pancreas | 7 |
| Testis | 4 |
| Breast | 4 |
| Heart | 2 |
| Skin | 2 |
| Diaphragm | 2 |

**Table III** Comparison of histology in prostate and metastases

| | *Surgical specimen versus lymph node metastases* | *Autopsy prostate*[a] *versus widespread metastases* |
|---|---|---|
| Similar histological pattern (same M.D. Anderson grade–MDAH) | 73.5% | 96%[b] |
| Metastases more differentiated (one or more MDAH grade difference) | 9.5% | 2% |
| Metastases less differentiated (one or more MDAH grade difference) | 16% | 2% |

[a]Available on 95 autopsies – 5 cases had prior radical prostatectomy; [b] 21% of the prostates had areas of differentiated tumour not present in the widespread metastases. The differentiated tumour was not sufficient in amount to alter the grade of the tumour.

**Table IV** Characteristics of nine patients who came to autopsy after staging lymphadenectomy

| *Years to autopsy* | *Histology of lymphadenectomy metastases* | *Histology of autopsy metastases* | *Radical prostatectomy* |
|---|---|---|---|
| 1 | Entirely differentiated | No metastases identified | Yes |
| 3 | Predominantly differentiated | Predominantly differentiated metastases in lymph nodes and bone | No |
| 4 | Entirely differentiated | No metastases identified | No |
| 4 | Predominantly differentiated | Widespread entirely undifferentiated | Yes |
| 5 | Predominantly differentiated | Widespread predominantly undifferentiated | No |
| 5 | Predominantly differentiated | Widespread predominantly undifferentiated | Yes |
| 6 | Entirely differentiated | Predominantly differentiated metastases in lymph nodes and bone | No |
| 6 | Entirely differentiated | Widespread entirely undifferentiated | Yes |
| 7 | Predominantly differentiated | Widespread entirely undifferentiated | No |

nuclear pyknosis, cytoplasmic vacuolisation and stromal fibrosis, but hormonal therapy has not been reported selectively to destroy differentiated areas of tumour (Franks, 1960). Further, tumour necrosis, suggestive of tumour destruction secondary to therapy, was not a conspicuous feature of any autopsy.

Another possibility is that undifferentiated metastases directly from the prostate are responsible for the widespread dissemination of metastatic prostate carcinoma. However, it appears that metastases can disseminate without input from the prostate. This concept is supported by the current study which included three cases which had autopsies after combined radical prostatectomy/staging lymphadenectomy. These three cases had entirely or predominantly differentiated metastases in the staging lymphadenectomies while the disseminated metastases at autopsy were entirely or predominantly undifferentiated. It is possible that some of the metastases in these three cases occurred before radical prostatectomy. However, in view of the widespread dissemination of these metastases it is unlikely that this accounts for all of the metastases. Further, the concept that metastases metastasise is in accordance with the cascade theory of metastases which has predicted that generalised metastases (such as brain metastases) do not ordinarily occur directly from the primary tumour (Viadana *et al.*, 1978).

The current study provides no support for the concept that differentiated metastases are restricted to pelvic lymph nodes while undifferentiated metastases spread to other sites. Autopsies with widespread metastases characteristically had similar undifferentiated histological patterns in all metastatic sites, including pelvic lymph nodes. Further, Butler *et al.* (1971) studied 19 patients who presented with metastases in enlarged supraclavicular lymph nodes as the initial presentation of previously unrecognised prostate carcinoma. The majority of the supraclavicular lymph node metastases were differentiated, which indicates that differentiated metastases are not restricted to pelvic lymph nodes.

It is unlikely that the inability to identify differentiated histological patterns in widespread metastases was due to sampling error. An average of 35 blocks of tissue were examined per autopsy. One-half of the blocks were usually from the prostate or from the metastases. The prostate not infrequently contained undifferentiated and differentiated histological patterns while the metastases characteristically revealed a monotonous pattern of undifferentiated tumour. It would be expected that differentiated histological patterns, if present in the widespread metastases, would be identified as easily as they were identified in the prostate itself.

It could be argued that the patients who came to autopsy represented a sub-population of patients with more aggressive disease than the lymphadenectomy cases and therefore were more likely to have undifferentiated patterns histologically. However, in the study of Butler *et al.* (1971) the majority of the 19 cases had differentiated supraclavicular lymph node metastases similar to the differentiated metastases depicted in Figure 1 of the current study. These 19 patients were compared to a control group of 100 patients who had prostate carcinoma but did not have a lymphadenectomy. The control group and the 19 patients had almost identical median survivals of 2 years 8 months and 2 years 11 months. Butler's data suggest that patients undergoing lymphadenectomy or patients whose initial lymph node metastases are differentiated do not represent a subpopulation of less aggressive prostate carcinoma.

Another explanation is that undifferentiated metastases destroy differentiated metastases. However, prostate carcinomas typically consist of multiple histological patterns. It is not widely believed that these histological patterns are in the process of destroying one another. An alternative explanation would be that a relative overgrowth of undifferentiated elements and cell loss of the differentiated components formed the undifferentiated patterns seen at autopsy. This explanation does not necessitate the destruction of differentiated metastases by undifferentiated metastases. However, Butler's study suggests: (1) that differentiated metastases are viable in locations other than pelvic lymph nodes; and (2) that differentiated metastases may obtain enough tumour bulk to enlarge supraclavicular lymph nodes. Consequently, it is unlikely that differentiated metastases would be easily overwhelmed or obscured by undifferentiated metastases.

The findings of the current study are most compatible with the concept that metastases dedifferentiate with time. The process of dedifferentiation has been demonstrated clinically and experimentally within primary tumours (Foulds, 1954; Kastendieck & Altenahr, 1976; Nowell, 1976;

Brawn, 1983; Barnett, 1984; Leonard & Smyth, 1985; Poste, 1986). It would not be unusual to expect a similar process to occur within metastases. Dedifferentiation obviates the need to hypothesise destruction of the initial differentiated metastases. Rather than being destroyed the initial differentiated metastases dedifferentiate and disseminate. Specific examples of the process of dedifferentiation are found in the current study, which identified five patients who had completely or predominantly differentiated metastases in staging lymphadenectomies and had completely or predominantly undifferentiated metastases at autopsy 4–7 years later.

The process of dedifferentiation presumably is a step by step process. This step by step process explains why some widespread metastases have residual differentiated histological patterns while the majority of widespread metastases are completely undifferentiated. The concept of histological dedifferentiation within metastases has clinical significance in that: (1) documenting the rapidity of dedifferentiation within metastases may allow one to predict the rapidity of dissemination of metastatic disease; and (2) slowing or halting dedifferentiation within metastases may control the dissemination of metastatic disease.

The authors thank Argye Hillis Ph.D, Director of Biostatistics, Scott and White Hospital, Temple, Texas, Associate Professor of Statistics, Texas A&M University for computing the statistical significance data, and Bobby Poff and Robert McEachern, Photographers at Texas A&M University School of Medicine, Veterans Administration Medical Center, Temple, Texas for photographic assistance.

### References

BARNETT, S.C. & ECCLES, S.A. (1984). Studies of mammary carcinoma metastasis in a mouse model system. *Clin. Exp. Metastases*, **2**, 15.

BRAWN, P.N. (1983). The dedifferentiation of prostate carcinoma. *Cancer*, **52**, 246.

BRAWN, P.N., AYALA, A.G., von ESCHENBACH, A.C., HUSSEY, D.H. & JOHNSON, D.E. (1982). Histologic grading study of prostate adenocarcinoma. *Cancer*, **49**, 525.

BRAWN, P.N. & JOHNSON, C.F. (1987). The metastatic potential of prostatic carcinomas composed entirely of single malignant glands. *Virchows Arch. A*, **411**, 399.

BRODERS, A.C. (1932). Practical points on the microscopic grading of carcinoma. *N.Y. J. Med.*, **32**, 667.

BUTLER, J.J., HOWE, C.D. & JOHNSON, D.E. (1971). Enlargement of the supraclavicular lymph nodes as the initial sign of prostatic carcinoma. *Cancer*, **27**, 1055.

FOULDS, L. (1954). The experimental study of tumour progression. *Cancer Res.*, **14**, 327.

FRANKS, L.M. (1960). Estrogen-treated prostatic cancer. *Cancer*, **13**, 490.

HADJI, M., TABEI, S.Z., CASTRO, A. & 4 others (1981). Prostatic specific antigen: An immunohistologic marker for prostatic neoplasms, *Cancer*, **48**, 1229.

KASTENDIECK, H. & ALTENAHR, E. (1976). Cyto and histomorphogenesis of prostate carcinoma. *Virchows Arch. A*, **370**, 207.

LEONARD, R.C.F. & SMYTH, J.F. (1985). The heterogeneity of human cancers. *Eur. J. Cancer Clin. Oncol.*, **21**, 1001.

MOSTOFI, F.K. (1975). Grading of prostatic carcinoma. *Cancer Chemother. Rep.*, **59**, 111.

NOWELL, P.C. (1976). The clonal evolution of tumour populations. *Science*, **194**, 23.

POSTE, G. (1986). Pathogenesis of metastatic disease. *Cancer Treat. Rep.*, **70**, 183.

SCARDINO, P.T. & CARLTON, C.E. (1983). Combined interstitial and external radiation for prostatic carcinoma. In *Principles and Management of Urologic Cancer*, 2nd edn p. 392. Williams and Williams: Baltimore.

VIADANA, E., BROSS, D.J. & PICKEN, J.W. (1978). In *Pulmonary Metastases*, Weiss, L. & Gilbert, H. (eds) p. 142. Hall: Boston.